# Feasibility of a Need‐Based Framework for Identifying Cognitive and Physical Needs in People Living with Cognitive Changes

**DOI:** 10.1002/alz70858_099922

**Published:** 2025-12-25

**Authors:** Shadi Gholizadeh, Amy Harris, Megan Harris

**Affiliations:** ^1^ TheKey (Formerly Home Care Assistance), San Diego, CA, USA; ^2^ TheKey Research Group, San Diego, CA, USA; ^3^ TheKey (Formerly Home Care Assistance), Omaha, NE, USA; ^4^ TheKey (Formerly, Home Care Assistance), San Diego, CA, USA

## Abstract

**Background:**

This study evaluated the feasibility of a need‐based framework to identify and address the cognitive (C) and physical (P) care needs of home care clients living with cognitive changes, including Alzheimer's disease and other dementias. Such lay‐friendly frameworks are particularly beneficial in settings where clinical diagnoses are unavailable or impractical. This initial implementation focused on creating a practical tool to support personalized care planning.

**Method:**

The study was conducted in four phases:

**Initial Stakeholder Feedback:**

Input was collected from professional caregivers, family members, client care managers, staffing managers, and general managers to refine the cognitive‐physical (C‐P) need framework.

**Pilot Testing:**

The framework was tested with clients across three offices [Nebraska (*N* = 12), Milwaukee (*N* = 7), and Sacramento (*N* = 7)]. Care managers rated clients using reverse numerical scales ranging from C4 (most severe cognitive needs, e.g., severe memory loss and disorientation) to C1 (least severe; e.g., mild forgetfulness) and P4 (most severe physical needs, e.g., total dependance for all daily tasks) to P1 (least severe, e.g., able to complete all personal care tasks without hands‐on support), demonstrating the diversity of care needs and the interplay between cognitive and physical requirements.

**Scientific Advisory Board (SAB) Review:**

The model was presented to a SAB including neurologists, geriatricians, and gero‐psychologists for feedback.

**Result:**

Findings from the pilot test revealed significant variability in client profiles, underscoring the framework's capacity to capture diverse care needs. Feedback from care teams highlighted the utility of the framework for settings where clinical diagnoses are unavailable. Based on the SAB recommendations, the framework was updated to include an emotional/behavioral (E) component, creating the C‐P‐E profile. Suggestions included expanding the list of example symptoms corresponding to the varying levels of physical and cognitive changes and reversing the numerical levels such that 1 indicates the highest severity.

**Conclusion:**

This need‐based framework provides a scalable tool for dementia care, with strong potential for enhancing care planning in non‐clinical settings. Future research will validate the expanded C‐P‐E framework, including psychometric testing for reliability and inter‐rater consistency, ensuring its applicability as a practical tool for diverse care environments.

